# Effects of Involved Laser Photons on Radiation and Electron-Positron Pair Production in one Coherence Interval in Ultra Intense Lasers

**DOI:** 10.1038/s41598-018-35312-8

**Published:** 2018-11-15

**Authors:** Bo Zhang, Zhi-meng Zhang, Zhi-gang Deng, Wei Hong, Jian Teng, Shu-kai He, Wei-min Zhou, Yu-qiu Gu

**Affiliations:** 1 Department of High Energy Density Physics, Research Center of Laser Fusion, 621900 Mianyang, Sichuan People’s Republic of China; 2 Laboratory of Science and Technology on Plasma Physics, Research Center of Laser Fusion, 621900 Mianyang, Sichuan People’s Republic of China

## Abstract

Electron radiation and *γ* photon annihilation are two of the major processes in ultra intense lasers (UIL). Understanding their behavior in one coherence interval (CI) is the basis for UIL-matter interaction researches. However, most existing analytic formulae only give the average over many CIs. Present understanding of these two multi-photon processes in one CI usually assume that they emit forward and their spectra have a cutoff at the energy of the electron/*γ*. Such assumptions ignore the effects of involved laser photons (EILP). We deduced the formulae for these two processes in one CI with EILP included and give the conditions for the EILP to be significant. Strong EILP introduces new behaviors into these two processes in one CI, such as large angle emission and emit particles above the usually assumed cutoff. Simulations show that the EILP would be significant when laser intensity reaches 2 × 10^22^ W/cm^2^, which is within the reach of state-of-art lasers.

## Introduction

Laser is presently the most intense electromagnetic field in laboratory. On next generation 10–100 petawatt laser facilities such as ELI^1^, laser intensity is anticipated to reach 10^23–25^W/cm^2^, which is several orders higher than the present record of 2 × 10^22^ W/cm^2^ ^2^. Such strong lasers will open a gate for many fundamental and fantastic strong field quantum electrodynamics (SFQED) phenomena^3–7^. For recent reviews, see^8–10^.

At such intensity, laser-matter interaction enters a new regime where two multi-photon SFQED processes, the quantum process of electron radiation (nonlinear Compton scattering, NCS)

1 $${e}^{-}(p)+n{\gamma }_{l}(k)\to \gamma (k^{\prime} )+{e}^{-}(p^{\prime} ),$$

and *γ* photon annihilation into an electron-positron pair (nonlinear Breit-Wheeler process, NBW)

2 $$\gamma (k^{\prime\prime} )+n^{\prime} {\gamma }_{l}(k)\to {e}^{-}(p^{\prime\prime} )+{e}^{+}(p\prime\prime\prime ).$$

become significant, i. e., can take a large fraction of laser energy (*p*, *k*, etc. are 4 vector momenta).

In ultra intense lasers, the CI of NCS and NBW $\delta \phi \sim 1/{a}_{0}\ll 1$ is very short, where the normalized field amplitude

3 $${a}_{0}\equiv \frac{e\sqrt{-{a}_{\mu }{a}^{\mu }}}{m}=\frac{e{E}_{L}}{m{k}_{0}},$$

*e* and *m* are electron charge and mass, *a*_*μ*_, *E*_*L*_ and *k*_0_ are the 4-vector, electric field and photon energy of the laser. Short CI means NCS and NBW happen in different CIs are independent from each other. NCS/NBW within a CI is then governed by the local field *F*_*μν*_ and instantaneous energy-momentum of the electron/*γ*.

Secondary interactions and back reactions are usually very strong in UILs, many emitted *γ* photons can produce *e*^−^ − *e*^+^ pairs and the produced charges can radiate, etc. NCSs can also change the electron and positron energy and momentum significantly within a CI. Due to short CI, strong secondary interactions and strong back reactions, the influence of NCS and NBW on UIL-matter interactions usually have to be investigated with simulations rather than analytic methods. In such simulations, NCS and NBW are treated as instantaneous and local processes.

Behavior of NCS/NBW in one CI is then the basis for UIL-matter interaction researches. Although NCS and NBW have already been studied extensively and a lot of references have discussed EILP, what most of these studies gave are the average over CIs of a period or a pulse with secondary interactions and back reactions ignored. Corresponding formulae cannot describe the behavior of NCS and NBW in one CI.

On the other hand, unfortunately, existing analytic studies of NCS and NBW within one CI only gave lightcone results. The electron emission probability^11^ in one CI in UILs (${a}_{0}\gg 1$) is

4 $$\begin{array}{rcl}\frac{d{N}_{NCS}}{dudt} & = & {F}_{\chi ,{p}_{0}}(u)\\  & = & \frac{\alpha }{\pi \sqrt{3}}\frac{{m}^{2}}{{p}_{0}}\frac{1}{{(1+u)}^{2}}[(1+u+\frac{1}{1+u}){K}_{2/3}(\frac{2u}{3\chi })\\  &  & -\,{\int }_{2u/3\chi }^{\infty }\,dy{K}_{1/3}(y)],\end{array}$$

where $\chi =e\sqrt{-{({F}_{\mu \nu }{p}^{\nu })}^{2}}/{m}^{3}$, ${K}_{\nu }(x)$ is the modified Bessel function of the *ν*th order, *α* is the fine structure constant, *p*_0_ is the electron energy and $u=kk^{\prime} /kp^{\prime}$. The pair production rate in one CI

5 $$\begin{array}{rcl}\frac{d{N}_{NBW}}{dtd\delta } & \approx  & \frac{\alpha {m}^{2}}{\sqrt{3}\pi {k^{\prime} }_{0}}[(\frac{1-\delta }{\delta }+\frac{\delta }{1-\delta }){K}_{2/3}(\kappa )\\  &  & -\,{\int }_{\kappa }^{\infty }\,{K}_{1/3}(y)dy].\end{array}$$

where $\chi ^{\prime} =e\sqrt{-{({F}_{\mu \nu }k{^{\prime\prime} }^{\nu })}^{2}}\,/\,{m}^{3}$, $\kappa =\frac{2}{3\chi \,^{\prime} \delta (1-\delta )}$ and $\delta =\frac{kp^{\prime\prime} }{kk^{\prime\prime} }$. These lightcone results are functions of ${k^{\prime} }_{0}-{\hat{e}}_{{\bf{k}}}\cdot {\bf{k}}^{\prime}$ and ${p^{\prime\prime} }_{0}-{\hat{e}}_{{\bf{k}}}\cdot {\bf{p}}{\boldsymbol{^{\prime\prime} }}$, respectively. Hence many important features of NCS and NBW in one CI such as spectrum and emission directions are obscure.

As an alternative, present UIL-matter interaction researches usually assume the *γ* photon emitted by NCS in one CI is along the instantaneous electron forward direction. The basis for this assumption on such a quantum process is that the emission angle of an ultra relativistic charge ~1/*γ* according to classical electrodynamics is very small^8,12,13^. The spectrum of NCS is also assumed to cut-off at the instantaneous electron energy *p*_0_. The basis for the second assumption is that, when the involved laser photon number *n* is small enough, considering the laser photon energy ${k}_{0}\ll {p}_{0}$, ${p}_{0}\approx {k^{\prime} }_{0}+{p^{\prime} }_{0}$ would be the upper limit for both ${k^{\prime} }_{0}$ and ${p^{\prime} }_{0}$.

Based on these two assumptions, present understanding of NCS in one CI usually take the emitted *γ* photon momentum ${\bf{k}}^{\prime} \approx u{\bf{p}}/(1+u)$ and the changed electron momentum ${\bf{p}}^{\prime} \approx {\bf{p}}/(1+u)$^14–16^. Similar assumptions for NBW give ${\bf{p}}^{\prime\prime} \approx \delta {\bf{k}}^{\prime\prime}$ and ${\bf{p}}{\boldsymbol{\prime\prime\prime }}\approx \mathrm{(1}-\delta ){\bf{k}}^{\prime\prime}$^14–16^.

Present understanding of NCS and NBW in one CI is a combination of the lightcone probabilities in Eqs 4 and 5 and the two assumptions. The model for NCS and NBW based on such understanding is widely accepted and applied in UIL-matter interaction researches^17–26^. As a consequence, existing simulation studies of UIL-matter interactions ignored EILP.

**Figure 1:**
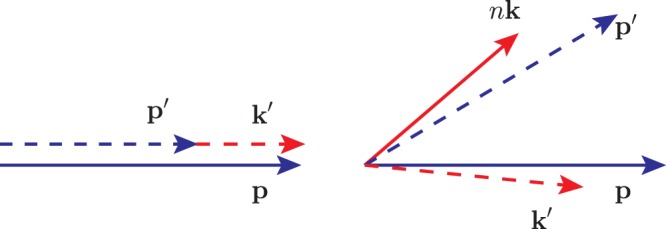
EILP and momentum conservation of NCS in a short CI in UILs. The left depicts present model and the right includes EILP.

However, the numbers of laser photons involved in these two processes, *n* and *n*′ scale as ~${a}_{0}^{3}$^8,11,27,28^, therefore the energy and momentum of involved laser photons grow nonlinearly with laser intensity. When laser intensity reaches *I* ~ 10^24^ W/cm^2^, the energy of laser photons involved in a single NCS *nk*_0_ or in a single NBW *n*′*k*_0_ would reach the scale of electron vibration energy *a*_0_*m* or the laser wake field acceleration record of 4.2 GeV^29^. According to energy and momentum conservation, the emission angle and spectrum of these two processes must be strongly influenced at such intensity. A simple diagram for EILP in NCS is shown in Fig. 1.

To investigate EILP in UIL-matter interactions, EILP on NCS and NBW in one CI is very important. The number of laser photons involved in a single NCS in UILs has a distribution peaked at

6 $${n}_{0}^{NCS}=\frac{u}{\chi }{a}_{0}^{3}.$$

Its dispersion ${\rm{\Delta }}n/{n}_{0}\sim {n}_{0}^{-1/3}$ is very small in UILs.

**Figure 2:**
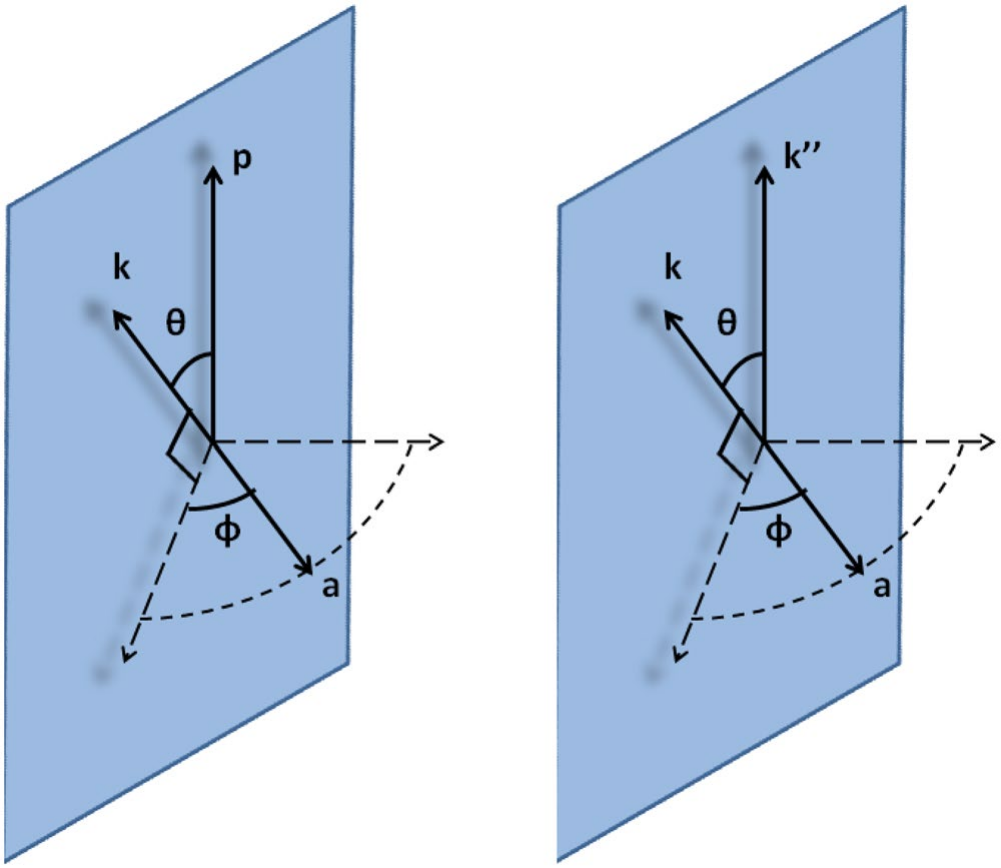
Geometry of NCS (left) and NBW (right) in general frames.

Without loss of generality, we put the electron momentum **p** on the *z* axis and **k** on *x*–*z* plane as shown in the left panel of Fig. 2. The angle between **p** and **k** is *θ* and the angle between **a** and **p** − **k** plane is $\phi$.

Solve the energy-momentum conservation equation of NCS, Eq. (6), the definition of *u* and an additional transverse distribution approximation $\tau /{a}_{0}=0$ simultaneously (see the supplemental information for details), where $\tau ={\tilde{F}}^{\mu \nu }{p^{\prime} }_{\mu }{p}_{\nu }/{m}^{2}{a}_{0}kk^{\prime}$, the momenta of the deflected electron and the emitted *γ* photon are

7 $$\begin{array}{rcl}{{\bf{p}}^{\prime} }_{\pm } & \approx  & \frac{(1+{u}^{2}C){p}_{0}}{1+u}\\  &  & (\begin{array}{c}\sin \,\theta \,{\sin }^{2}\,\frac{{\theta }_{p^{\prime} }^{B}}{2}\pm \,\sin \,\frac{\theta }{2}\,\cos \,\phi \,\sin \,{\theta }_{p^{\prime} }^{B}\\ \mp \sin \,\frac{\theta }{2}\,\sin \,{\theta }_{p^{\prime} }^{B}\,\sin \,\phi \\ 1-2\,{\sin }^{2}\,\frac{{\theta }_{p^{\prime} }^{B}}{2}\,{\sin }^{2}\,\frac{\theta }{2}\pm \,\cos \,\phi \,\cos \,\frac{\theta }{2}\,\sin \,{\theta }_{p^{\prime} }^{B}\end{array})\end{array}$$

and

8 $$\begin{array}{rcl}{{\bf{k}}^{\prime} }_{\pm } & \approx  & \frac{u(1+C){p}_{0}}{1+u}\\  &  & (\begin{array}{c}\sin \,\theta \,{\sin }^{2}\,\frac{{\theta }_{k^{\prime} }^{B}}{2}\mp \,\sin \,\frac{\theta }{2}\,\cos \,\phi \,\sin \,{\theta }_{k^{\prime} }^{B}\\ \pm \sin \,\frac{\theta }{2}\,\sin \,{\theta }_{k^{\prime} }^{B}\,\sin \,\phi \\ 1-2\,{\sin }^{2}\,\frac{{\theta }_{k^{\prime} }^{B}}{2}\,{\sin }^{2}\,\frac{\theta }{2}\mp \,\cos \,\phi \,\cos \,\frac{\theta }{2}\,\sin \,{\theta }_{k^{\prime} }^{B}\end{array}),\end{array}$$

where

9 $$\begin{array}{c}\cos \,{\theta }_{p^{\prime} }^{B}\approx \frac{1-{u}^{2}C}{1+{u}^{2}C}\\ \cos \,{\theta }_{k^{\prime} }^{B}\approx \frac{1-C}{1+C}\end{array}$$

and $C={a}_{0}^{3}{k}_{0}/\chi {p}_{0}$. The ± subscripts are the signs of

10 $$\rho =\frac{e{F}^{\mu \nu }{p^{\prime} }_{\mu }{p}_{\nu }}{{m}^{2}{a}_{0}^{2}kk^{\prime} },$$

The chances for + and − are equal because the differential probability is a even function of $\rho$^11^. Emitted photon energy and the emission angle are

11 $$\begin{array}{rcl}\cos \,{\theta }_{k^{\prime} \pm } & = & \frac{1-2\,{\sin }^{2}\,\frac{{\theta }_{k^{\prime} }^{B}}{2}\,{\sin }^{2}\,\frac{\theta }{2}\mp \,\cos \,\phi \,\cos \,\frac{\theta }{2}\,\sin \,{\theta }_{k^{\prime} }^{B}}{1\mp \,\cos \,\phi \,\cos \,\frac{\theta }{2}\,\sin \,{\theta }_{k^{\prime} }^{B}}\\ {k^{\prime} }_{0,\pm } & = & \frac{u(1+C)}{1+u}{p}_{0}(1\mp \,\cos \,\phi \,\cos \,\frac{\theta }{2}\,\sin \,{\theta }_{k^{\prime} }^{B}).\end{array}$$

**Figure 3:**
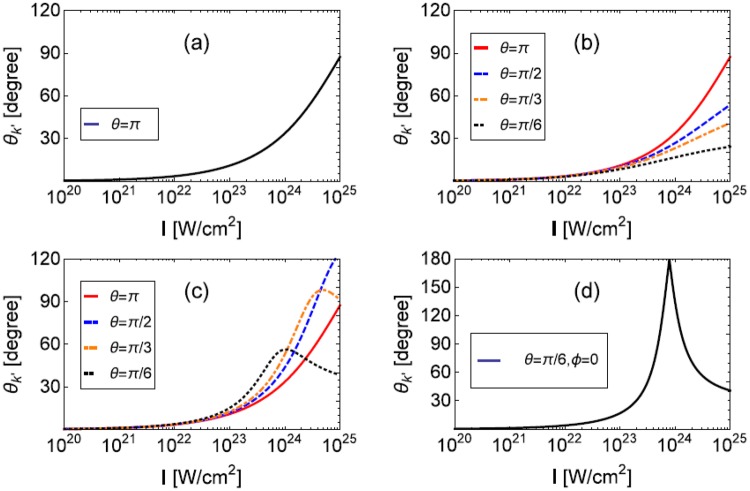
Emission angle of NCS in one CI, *θ*_*k*′±_ when **k** and **p** are antiparallel (**a**), *θ*_*k*′+_ when $\phi =\pi /6$ (**b**), *θ*_*k*′−_ when $\phi =\pi /6$ (**c**) and *θ*_*k*′−_ when $\theta =\pi /6$ and $\phi =0$ (**d**). Corresponding parameters are ${p}_{0}=1.022\,{\rm{GeV}}$, ${k}_{0}=1.24\,{\rm{eV}}$ (*λ* = 1 *μ*m) and $I={a}_{0}^{2}1.37\times {10}^{18}$ W/cm^2^.

Figure 3 shows the emission angle of a 1 GeV electron when it radiates at the peak of an UIL. Figure 3(a) is the head-on case, it is larger than 30° at *I* = 10^24^ W/cm^2^. Figure 3(b,c) show emission angles *θ*_*k*′+_ and *θ*_*k*′−_ when $\phi =\pi /6$. Figure 3(d) gives the emission angle when **k**, **p** and **a** are on the same plane. It shows that when $\phi$ is small, the emission angle can be very large. This is quite different from present understanding of NCS in one CI in UILs where forward emission (vanishing emission angle) is assumed^14–26^.

This non-vanishing fixed emission angle with respect to instantaneous electron momentum is different from that in^27^ which is with respect to the electron momentum at infinity where laser vanishes, i. e., before it enters the laser. Additionally, the emission angle in one CI is fixed in arbitrary frames while that in ref.^27^ is fixed only in specific frames that **k** and the electron momentum at infinity are anti-parallel. This angle is also different from those induced by stochastic effects and consecutive radiations in refs^24,26^.

**Figure 4:**
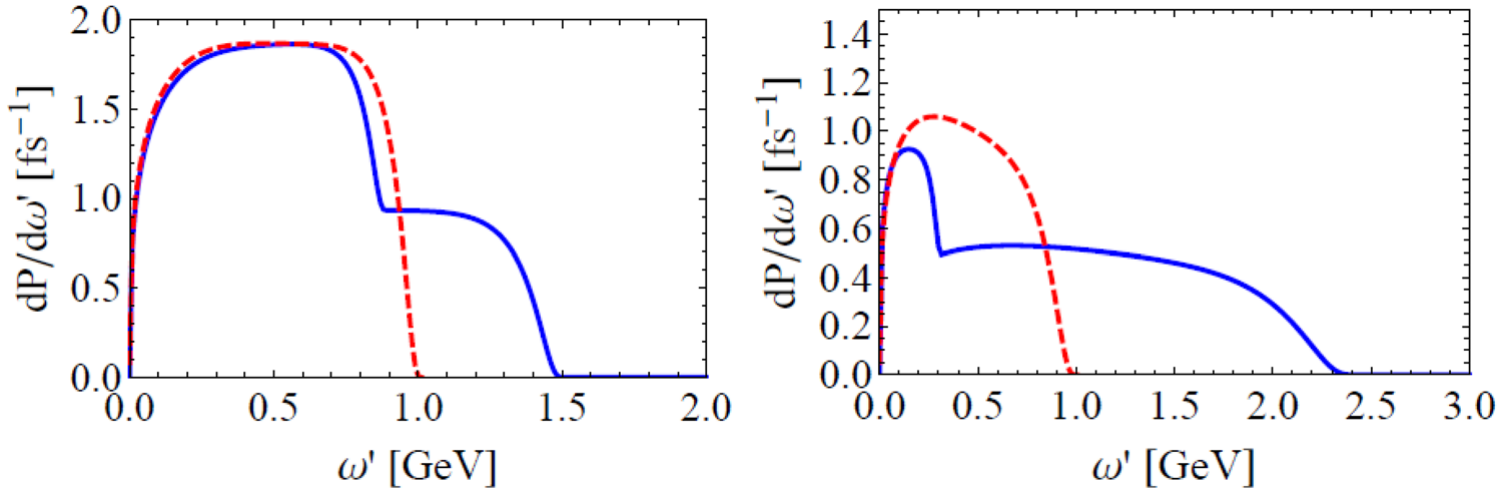
Energy integrated spectrum of a 1 GeV electron including and excluding EILP (blue solid, red dashed). $I={10}^{24}$ W/cm^2^, $\theta =\pi /2$, $\phi =\pi /3$ for the left panel and $\theta =\pi /3$, $\phi =0$ for the right.

The spectrum of the emitted photon is also modified by EILP. Comparison of energy integrated spectra $dP/d\omega ^{\prime} dt=\omega ^{\prime} dN/d\omega ^{\prime} dt$ of a 1 GeV electron at the moment it appears at the peak of an UIL of *I* = 10^24^ W/cm^2^ including EILP and excluding it is shown in Fig. 4. As given in Eq. (11), EILP blue shifts half of the spectrum and red shifts the other half. Hence the spectrum gets a two-stage structure and the blue shifted part can surpass the instantaneous electron energy of *p*_0_ = 1 GeV, which is the cutoff in present model of single NCS in UILs.

EILP has similar influences on NBW in one CI. The involved laser photon number *n*′ is approximately

12 $${n^{\prime} }_{0}=\frac{1}{\delta (1-\delta )\chi ^{\prime} }{a}_{0}^{3}$$

and its dispersion ${\rm{\Delta }}n^{\prime} /{n^{\prime} }_{0}$ is of ${({n^{\prime} }_{0})}^{-\mathrm{1/3}}$ scale. As shown in the right panel of Fig. 2, without loss of generality, we fix the *γ* photon momentum **k″** on the *z* axis and **k** on *x*–*z* plane. The angle between **k**″ and **k** is *θ* and the angle between **a** and **k**″ − **k** plane is $\phi$.

Similar to the NCS case, solve the energy-momentum conservation of NBW, Eq. (12), definition of *δ* and again the additional approximation $\delta /{a}_{0}=0$ simultaneously (see the supplemental information for details), the emitted electron momentum is

13 $$\begin{array}{rcl}{{\rm{p}}^{\prime} }_{\pm } & = & \frac{({k^{\prime} }_{0}+n{k}_{0})}{2}(1+(2\delta -1)\zeta )\\  &  & (\begin{array}{c}\sin \,\theta \,{\sin }^{2}\,\frac{{\theta }_{p^{\prime\prime} }^{B}}{2}\mp \,\sin \,\frac{\theta }{2}\,\cos \,\phi \,\sin \,{\theta }_{p^{\prime\prime} }^{B}\\ \pm \sin \,\frac{\theta }{2}\,\sin \,{\theta }_{p^{\prime\prime} }^{B}\,\sin \,\phi \\ 1-2\,{\sin }^{2}\,\frac{{\theta }_{p^{\prime\prime} }^{B}}{2}\,{\sin }^{2}\,\frac{\theta }{2}\mp \,\cos \,\frac{\theta }{2}\,\cos \,\phi \,\sin \,{\theta }_{p^{\prime\prime} }^{B}\end{array}),\end{array}$$

and the emitted positron momentum is

14 $$\begin{array}{rcl}{{\rm{p}}^{\prime\prime} }_{\pm } & = & \frac{({k^{\prime\prime} }_{0}+n{k}_{0})}{2}\mathrm{(1}-\mathrm{(2}\delta -\mathrm{1)}\zeta )\\  &  & (\begin{array}{c}\sin \,\theta \,{\sin }^{2}\,\frac{{\theta }_{p\prime\prime\prime }^{B}}{2}\pm \,\sin \,\frac{\theta }{2}\,\cos \,\phi \,\sin \,{\theta }_{p\prime\prime\prime }^{B}\\ \mp \sin \,\frac{\theta }{2}\,\sin \,{\theta }_{p\prime\prime\prime }^{B}\,\sin \,\phi \\ 1-2\,{\sin }^{2}\,\frac{\theta }{2}\,{\sin }^{2}\,\frac{{\theta }_{p\prime\prime\prime }^{B}}{2}\pm \,\cos \,\phi \,\cos \,\frac{\theta }{2}\,\sin \,{\theta }_{p\prime\prime\prime }^{B}\end{array}),\end{array}$$

where

15 $$\begin{array}{c}\cos \,{\theta }_{p^{\prime\prime} }^{B}=\frac{\zeta +(2\delta -1)}{1+(2\delta -1)\zeta }\\ \cos \,{\theta }_{p\prime\prime\prime }^{B}=\frac{\zeta -(2\delta -1)}{1-(2\delta -1)\zeta },\end{array}$$

and

16 $$\zeta =\frac{{k^{\prime\prime} }_{0}-n{k}_{0}}{{k^{\prime\prime} }_{0}+n{k}_{0}}.$$

Again, ± subscripts are the signs of

17 $$\rho ^{\prime} =\frac{e{F}^{\mu \nu }{p\prime\prime\prime }_{\mu }{p^{\prime\prime} }_{\nu }}{{m}^{2}{a}_{0}^{2}kk^{\prime\prime} }$$

and both signs take half of the chance.

**Figure 5:**
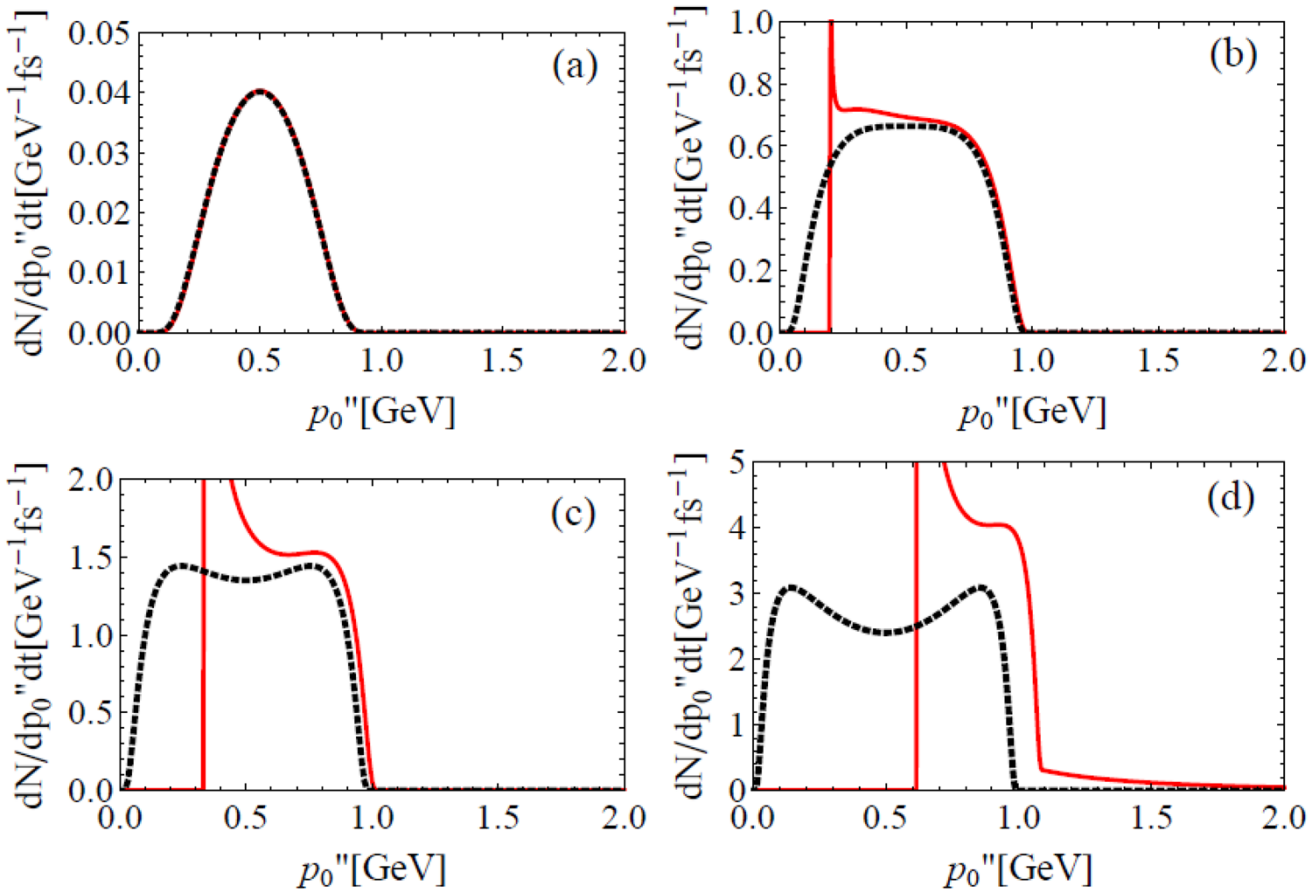
EILP on the spectrum $d{N}_{NBW}/d{p^{\prime\prime} }_{0}dt$ of electron produced by NBW (red lines). The parameters are: $\theta =\pi$, $\phi =0$, ${k^{\prime\prime} }_{0}=1$ GeV, ${k}_{0}=1.24$ eV (*λ* = 1 *μ*m) and $I={a}_{0}^{2}1.37\times {10}^{18}$ W/cm^2^ is 10^22^ (**a**), 10^23^ (**b**), 3 × 10^23^ (**c**) and 10^24^ W/cm^2^ (**d**), respectively. For comparison, spectra given by present NBW model (black dotted) were also shown.

EILP on a NBW in UILs is similar but different. When laser intensity is comparatively low, the *e*^−^ spectrum with EILP agrees with the present model as Fig. 5(a) shows, note that the spectrum of *e*^+^ is the same. With the growth of laser intensity, as shown in (b) and (c), the EILP introduces a lower limit and this is due to the minimal number of laser photons involved in a NBW that can be easily deduced from Eq. (12). Finally, when laser intensity is very high, as (d) shows, the cutoff at ${k^{\prime\prime} }_{0}$ disappears.

**Figure 6:**
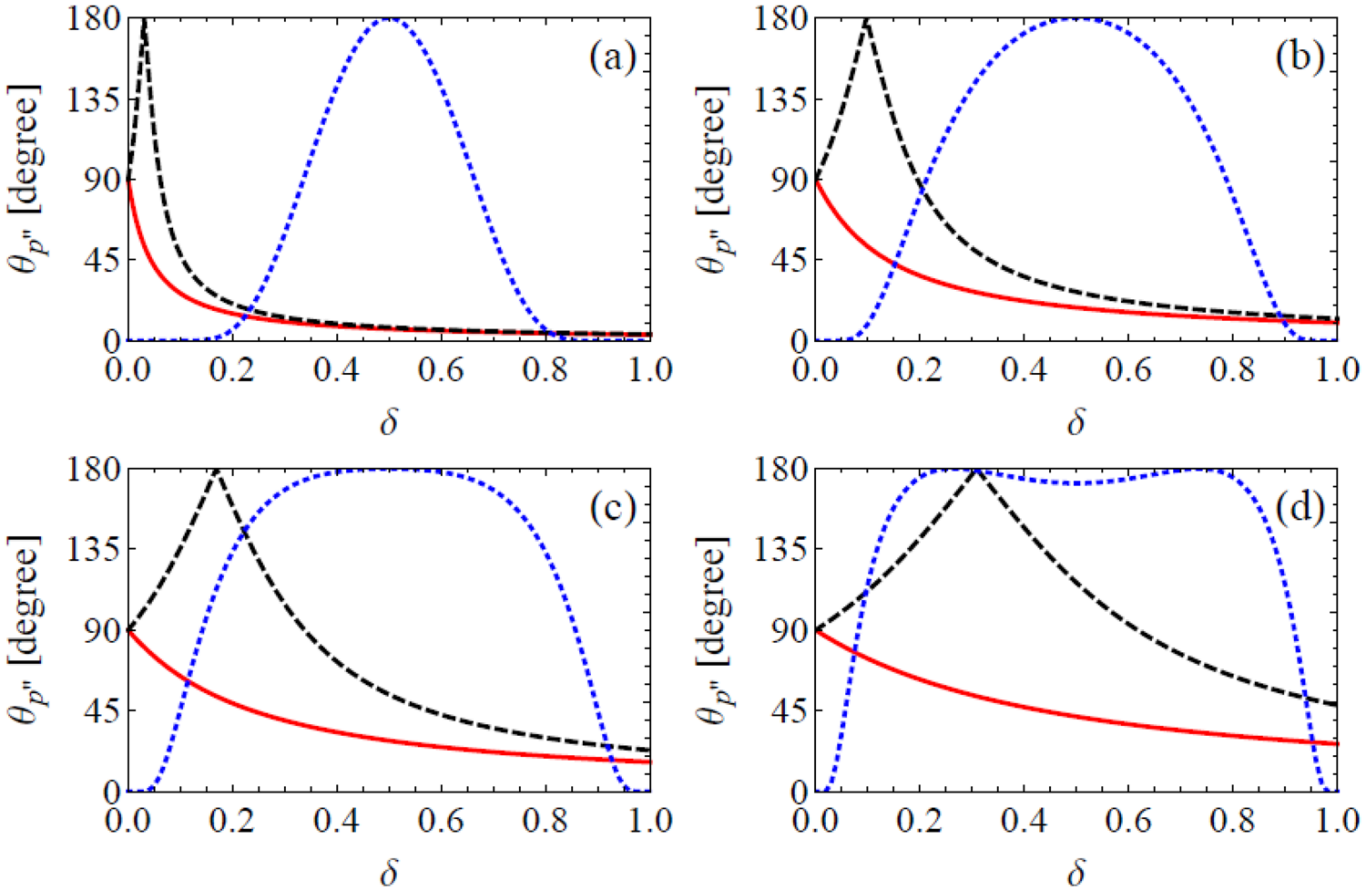
Emission angle *θ*_*p*″±_ (red solid line for + and black dashed for −) of NBW with respect to instantaneous *γ* photon momentum **k**″ in UILs. Parameters are: $\theta =\pi /2$, $\phi =0$, ${k^{\prime\prime} }_{0}=1$ GeV, ${k}_{0}=1.24$ eV (*λ* = 1 *μ*m) and $I={a}_{0}^{2}1.37\times {10}^{18}$ W/cm^2^ is 10^22^ (**a**), 10^23^ (**b**), 10^24^ (**c**) and 10^25^W/cm^2^ (**d**), respectively. The blue dotted lines are normalized differential probabilities.

Different from the NCS case, the non vanishing emission angle of NBW in one CI introduced by EILP is not fixed. Figure 6 shows the emission angle *θ*_*p*″±_ of *e*^−^ in UILs, it strongly depends on *δ*. Note that the emission angle of *e*^+^ is symmetric with respect to *δ* = 0.5. The differential probabilities are also present to show the physical significant ranges of *δ*. When laser intensity is low, *e*^−^ and *e*^+^ are highly possible to be emitted along directions close to **k**″. The probability of large angle *e*^−^ and *e*^+^ emission grows with laser intensity, and becomes dominant when $I\gtrsim {10}^{24}$ W/cm^2^.

To explore signals of EILP on NCS and NBW on future laser facilities, Monte-Carlo simulations of interactions between an electron bunch and UIL pulses are carried out. Eqs (4), (7) and (8) are employed to describe NCSs and NBWs including EILP are described by Eqs (5), (13) and (14). Between emissions, classical equations of motion are applied to describe electron propagation. Other processes such as higher order radiations^30^ are neglected for their much weaker effects under the laser conditions concerned.

**Figure 7:**
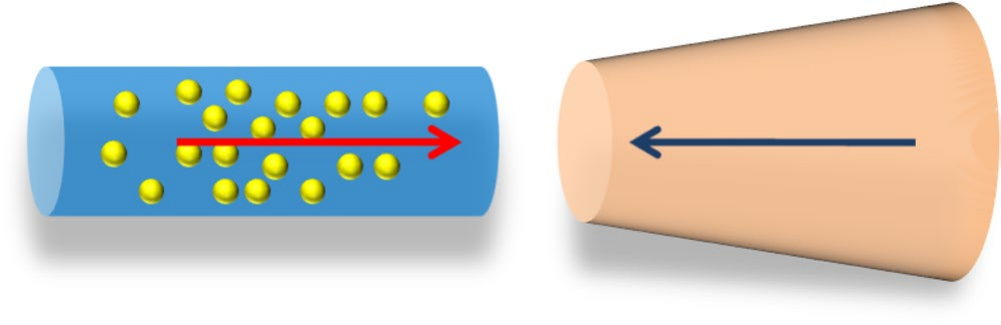
Schematics of the simulations.

In the simulation, as shown in Fig. 7, a 1.022 GeV quasi mono-energetic electron bunch collides head-on with a tightly focused linearly polarized short laser pulse. The laser field is given by an approximate solution of Maxwell’s equations to the first order of ${(|{\bf{k}}|{w}_{0})}^{-1}$ and ${({\omega }_{0}{\tau }_{0})}^{-1}$, where *w*_0_ and *τ*_0_ are the waist radius and pulse duration^31^. Electromagnetic force between electrons is ignored for it is 7–8 magnitudes weaker than the laser Lorentz force.

Applied parameters are the following: peak intensity of the laser pulse is ${I}_{0}={E}_{{\max }}^{2}/2=2\times {10}^{22}$ and 10^24^ W/cm^2^, the laser wave length *λ* = 1 *μ*m, beam waist *w*_0_ = 1 *μ*m and duration ${\tau }_{0}=2\lambda /c=6.7$ fs. The electron bunch includes $N={10}^{6}$ electrons, which uniformly distribute in a *R* = 1 *μ*m sphere. The mean initial electron energy is 1.022 GeV (${\gamma }_{0}=2000$), and both the energy dispersion ${\rm{\Delta }}\gamma /{\gamma }_{0}$ and angular dispersion ${\rm{\Delta }}$*θ* are 0.001. The initial longitudinal distance between the electron bunch and laser pulse centers is 20 *μ*m, the simulation lasts 330 fs, which allows most particles to escape the pulse. The time step is ${\rm{\Delta }}t=1.67\times {10}^{-2}$ fs, which is fine enough for the results to converge.

**Figure 8:**
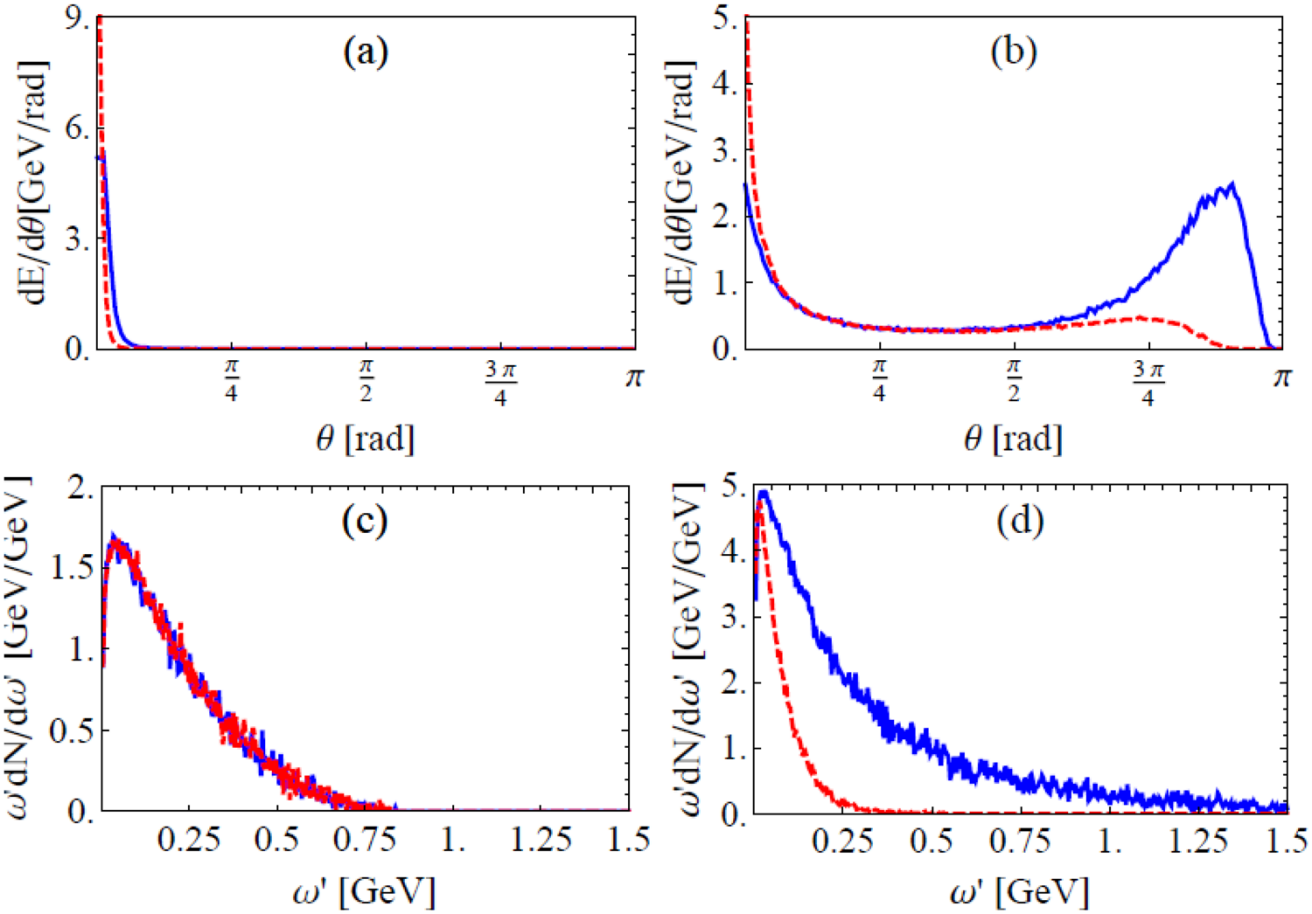
Angular distribution of radiation intensity when ${I}_{0}=2\times {10}^{22}$ W/cm^2^ (**a**) and 10^24^ W/cm^2^ (**b**) and energy integrated spectra of emitted photons for ${I}_{0}=2\times {10}^{22}$ (**c**) and 10^24^ W/cm^2^ (**d**), (**d**) only includes photons in the backward hemisphere of initial electron bunch. All normalized by the initial electron number *N*_*e*_, blue solid lines include EILP and red dashed lines exclude EILP.

Simulation results of the ${I}_{0}={E}_{max}^{2}/2=2\times {10}^{22}$ W/cm^2^ case are shown in Fig. 8(a,c). Radiation intensity angular distribution of emitted *γ* photons including and excluding EILP both concentrate around the initial electron bunch direction, EILP extends the angular divergence (FWHM) from 2° to 4°. Note it is different from that induced by consecutive stochastic emissions^24^,

When the laser intensity is increased to 10^24^ W/cm^2^, as shown in Fig. 8(b), EILP creates a strong, new peak in emission intensity angular distribution which is very close to the laser forward direction. Note that the mechanism for this peak is different from the stochastic effect peak in^26^ and the parameters are quite different (${\gamma }_{0}\gg 2{a}_{0}$ in our case while ${\gamma }_{0} < 2{a}_{0}$ in ref.^26^). EILP on the photon spectrum is also significant. Figure 8(d) shows that, the spectrum of photons emitted in the backward hemisphere excluding EILP is bounded below 0.5 GeV while that including EILP extends 1.5 GeV and higher, and the intensity of radiation is also several times higher.

In conclusion, EILP on NCS and NBW in one CI in UILs is investigated. Present understanding of these two processes is based on two assumptions, forward emission and spectrum cutoff. These assumptions that exclude EILP are good approximations when the energy scale of involved laser photon ${a}_{0}^{3}{k}_{0}$ is much lower than that of the electron/*γ* photon.

When the total energy of involved laser photons becomes comparable to that of the electron/*γ* photon, NCS/NBW can have large emission angles and the high energy part of spectrum can surpass the instantaneous electron/*γ* photon energy. Furthermore, the spectrum of NBW gets an additional lower limit.

Simulation results demonstrate that EILP is very important in future SFQED experiments, it dominates single NCS/NBW when laser intensity is close to 10^24^ W/cm^2^. Its effects is significant at a much lower intensity of 2 × 10^22^ W/cm^2^, which is within the reach of state-of-art lasers.

## Electronic supplementary material

Supplemental Information

## Data Availability

The data that support the findings of this study are available from the corresponding authors on reasonable request.
