# Supplementary material for: Effects of Involved Laser Photons on Radiation and Electron-Positron Pair Production in one Coherence Interval in Ultra Intense Lasers
Source: Sci Rep. 2018 Nov 15;8:16862. doi: 10.1038/s41598-018-35312-8 (PMC6237868; doi:10.1038/s41598-018-35312-8)
Supplement: Supplementary file 1 — Supplemental Information [file 41598_2018_35312_MOESM1_ESM.pdf]

Supplementary Information for "Effects of Involved Laser Photons on Radiation and Electron-Positron Pair Production in one Coherence Interval in Ultra Intense Lasers"

Bo Zhang,[1,2] Zhi-meng Zhang,[1,2] Zhi-gang Deng,[1] Wei Hong,[1,2]
Jian Teng,[1,2] Shu-kai He,[1] Wei-min Zhou,[1,2] and Yu-qiu Gu[1,2]

[1] *Department of High Energy Density Physics, Research Center of Laser Fusion, 621900, Mianyang, Sichuan, People's Republic of China*
[2] *Laboratory of Science and Technology on Plasma Physics, Research Center of Laser Fusion, 621900, Mianyang, Sichuan, People's Republic of China*

(Dated: October 29, 2018)

The coherence length of nonlinear Compton scattering (NCS)

$$e^-(p) + n\gamma_l(k) \to \gamma(k') + e^-(p'), \tag{1}$$

and nonlinear Breit-Wheeler (NBW) process

$$\gamma(k'') + n\gamma_l(k) \to e^-(p'') + e^+(p'''). \tag{2}$$

in an ultra intense laser (UIL) where $a_0 \gg 1$ is $\Delta\phi \sim 1/a_0$. NCS and NBW are therefore instantaneous and local processes in UILs. Then the external field the electron/γ photon feels in a NCS/NBW is approximately a constant crossed field (CCF) and the number of laser photons involved in this NCS/NBW process is determined by the instantaneous energy-momentum p/k'' of the electron/γ photon and the local CCF. Since NCS and NBW are symmetric processes, we focus on the number of laser photons involved in NCS in this supplemental material and give the NBW results at the end.

It is a well known conclusion in strong field quantum electrodynamics (SFQED) that the number n of laser photons an electron involved in a single NCS in UIL is of the order of a_0^3. However, to investigate the effect of involved laser photons (EILP) in NCS, the qualitative relation $n \sim a_0^3$ is far from enough, a quantitative n is needed.

A physically correct differential probability W of NCS should be Lorentz and gauge invariant. Hence it is a function of corresponding invariant parameters of the initial state. The initial state has 3 Lorentz vectors, a^μ, k^μ and p^μ. Their combinations give 4 independent non vanishing parameters: χ, a_0

$$\begin{aligned} a_0 &= \frac{e\sqrt{-a_\mu a^\mu}}{m} \\ \chi &= \frac{e\sqrt{-(F_{\mu\nu}p^\nu)^2}}{m^3} \\ f &= \frac{e^2 F^{\mu\nu}F_{\mu\nu}}{4m^4} \\ g &= \frac{e^2 F^{\mu\nu}\tilde{F}_{\mu\nu}}{4m^4}. \end{aligned} \tag{3}$$

Among these 4 parameters, f and g vanish in a CCF therefore χ and a_0 distinguish physically different initial states of NCS in CCF that cannot be connected by Lorentz transformations. Thus they determine the differential probability W^{CCF}, i. e., $W^{CCF} = W^{CCF}_{\chi,a_0}$.

A set of independent Lorentz and gauge invariant parameters for the production of NCS includes the scattered photon number n, u and

$$\tau = \frac{\tilde{F}^{\mu\nu}p'_\mu p_\nu}{m^2 a_0 kk'}. \tag{4}$$

Hence $W^{CCF} = W^{CCF}_{\chi,a_0}(n, u, \tau)$. Note that n is not predetermined therefore is a parameter of NCS production.

Considering the NCS in a linearly polarized plane wave field concentrates around $|\tau| \lesssim 1$ when $a_0 \gg 1$, the deviation of radiated photon from the $\mathbf{k}-\mathbf{a}-\mathbf{p}$ plane $\sim \tau/a_0 \to 0$ is strongly suppressed in the frames where $\mathbf{k}$ and $\mathbf{p}$ are antiparallel [1]. Hence the differential probability is approximately $W^{CCF}_{\chi,a_0}(n,u)\delta(\tau)$ in strong field limit.

Our method to determine $W^{CCF}_{\chi,a_0}(n,u)$ is to extract this number from corresponding circularly polarized (CP) plane wave formulae. NCS in CP plane waves can be considered as the scattering of dressed electrons with γ photons. Momentum conservation in this case is replaced by conservation of quasi-4-momentum ($\mu = 0$ for energy to $1-3$ for momentum, e. g., $k = (k_0, \mathbf{k})$)

$$q^\mu + n^{CP}k^\mu = q'^\mu + k'^\mu, \tag{5}$$

where

$$\begin{aligned} q^\mu &= p_I^\mu + \frac{m^2 a_0^2}{2kp}k^\mu \\ q'^\mu &= p_I'^\mu + \frac{m^2 a_0^2}{2kp'}k^\mu \end{aligned} \tag{6}$$

are quasi-momenta of the electron before and after scattering in CP plane wave field and n^{CP} the quasi scattered photon number. Note that p_I^μ and $p_I'^\mu$ are momenta at infinity where the field vanishes.

The differential probability W^{CP} was given in Ref. [1–3] as

$$\begin{aligned} &\frac{\partial W^{CP}_{n^{CP}}}{\partial u} \\ &= \frac{e^2m^2}{4q_0}\frac{1}{(1+u)^2}[-4J_n^2(z_n(u)) + a_0^2(\frac{u^2+2u+2}{1+u}) \\ &\quad (J_{n+1}^2(z_n) + J_{n-1}^2(z_n) - 2J_n^2((z_n))], \end{aligned} \tag{7}$$

where

$$y_n = \frac{2nkp}{m_*^2}$$
$$m_*^2 = m^2(1+a_0^2)$$
$$z_n(u) = \frac{2a_0}{y_1}\sqrt{\frac{u(y_n-u)}{1+a_0^2}}. \quad (8)$$

and J_n is Bessel function of the first kind.

When $a_0 \gg 1$,due to short coherence length, the differential probability W^{CP} of NCS in a CP plane wave is the average of single NCSs differential probability $W^{CCF}(\phi)$ in the local constant CCF of UILs over a period.

The local field $F^{CCF} = F^{CP}(\phi)$ and the instantaneous electron momentum $p(\phi, a_0, p_0^\mu)$ is

$$p_\perp(\phi) = ea(\phi) + p_{\perp I}$$
$$p^0(\phi) - p_\parallel(\phi) = p_I^0 - p_{\parallel I} = \sigma$$
$$p_\parallel(\phi) = \frac{m^2 - \sigma^2 + p_\perp(\phi)^2}{2\sigma}$$
$$p^0(\phi) = \frac{m^2 + \sigma^2 + p_\perp(\phi)^2}{2\sigma} \quad (9)$$

where $p_\parallel$ is the projection of $\mathbf{p}$ along $\mathbf{k}$ direction and $p_\perp = p - p_\parallel$.

Put $a^\mu(\phi)$ and $p(\phi)$ into Eq. (3), both $a_0(\phi)$ and $\chi(\phi)$ are independent from ϕ, hence the differential probability

$$W^{CCF}_{\chi(\phi),a_0(\phi)}(n,u) = W^{CCF}_{\chi,a_0}(n,u), \quad (10)$$

is the same over a period.

Further consider that the quasi-momentum q and q' are the average of $p(\phi)$ and $p'(\phi)$, i. e.,

$$q = \int_0^{2\pi} d\phi p(\phi)$$
$$q' = \int_0^{2\pi} d\phi p'(\phi), \quad (11)$$

the involved laser photon number n in the local CCF is the same to that in corresponding CP plane wave, i. e.,

$$n^{CCF}_{NCS} = n^{CP}. \quad (12)$$

Note that n^{CCF}_{NCS} is approximately the same to n^{CP} in this limit but have different physical meanings. The former is based on momentum conservation and is about a single NCS while the later is based on quasi-momentum conservation and the average of single NCSs over a period.

Fortunately, n^{CP} has a narrow distribution. In the $J_n(z)$ appeared in Eq. (7), z distributes from 0 to $na_0/\sqrt{1+a_0^2} \approx n$. In this region, $J_n(z)$ in large n limit is highly suppressed except when $z \approx n$ [4]. Since both terms of Eq. (7) are only significant when $n - z \lesssim n^{1/3}$, the significant part of W^{CP} comes from regions where $u \approx y_n/2$, or more specifically,

$$|u - y_n/2| \lesssim \frac{y_n}{n^{1/3}}. \quad (13)$$

In this narrow region,

$$\frac{u}{\chi}a_0^3 = \frac{n^{CP}}{2}(1 - a_0^{-2} + \mathcal{O}(n^{-1/3})). \quad (14)$$

In other words, the distribution of n^{CP} is centered around

$$n_0^{CP} = \frac{u}{\chi}a_0^3 \quad (15)$$

and the divergence

$$\Delta n^{CP}/n_0^{CP} \sim a_0^{-1} \quad (16)$$

vanishes in large a_0 limit. Then the differential probability W^{CCF} can be further simplified to

$$W^{CCF}_{\chi,a_0}(n,u,\tau) \approx W^{CCF}_{\chi,a_0}(u)\delta(n - \frac{u}{\chi}a_0^3)\delta(\tau). \quad (17)$$

Since the "u spectrum" $F_{\chi,a_0}(u)$ in CCF is known [1] as Eq. (3) of the paper, we can arrive at the strong field approximation for the differential probability of a single NCS in local CCF of an UIL as

$$W^{CCF}_{\chi,a_0}(n^{CCF}_{NCS},u) \approx F_{\chi,a_0}(u)\delta(n^{CCF}_{NCS} - \frac{u}{\chi}a_0^3). \quad (18)$$

Note the relation $n \sim \mathcal{O}(a_0^3)$ is also recovered since u/χ is of the scale of 1.

Then we obtain the equations for single NCS:

$$\begin{cases} nk + p = k' + p' \\ u = \dfrac{kk'}{kp'} \\ n = \dfrac{u}{\chi}a_0^3 \\ \tau/a_0 = 0 \end{cases}, \quad (19)$$

where the first line is energy-momentum conservation. The last line comes from $\tau/a_0 \ll 1$ in large a_0 limit [1], it bounds the radiation close to the $\mathbf{k} - \mathbf{a} - \mathbf{p}$ plane in frames where $\mathbf{k}$ and $\mathbf{p}$ are anti-parallel.

We fist solve these equations in the simplest case where $\mathbf{k}$ and $\mathbf{p}$ are anti-parallel.

In this case, it is easy to get the radiated γ photon energy and the emission angle are

$$k_0'^A \approx \frac{u(1+C)}{1+u}p_0$$
$$\cos\theta^A_{k'} \approx \frac{1-C}{1+C} \quad (20)$$

where $C = a_0^3 k_0/\chi p_0$, $\theta^A_{k'}$ is the angle between $\mathbf{k}'$ and $\mathbf{p}$ in this case and A denotes the antiparallel case. Apparently, the two solutions for $\mathbf{k}'$ with different signs of ρ distribute symmetrically with respect to $\mathbf{p}$ in this frame.

The corresponding energy and deflection angle of the electron after the scattering are

$$p_0'^A \approx \frac{1+u^2C}{1+u}p_0$$
$$\cos\theta^A_{p'} \approx \frac{1-u^2C}{1+u^2C} \quad (21)$$

Note that $\theta^A_{k'}$ is independent of u while $\theta^A_{p'}$ is not, which means the emission angle is fixed while the deflection angle is not.

Results obtained in this case can be extended to arbitrary frames through Lorentz transformation shown in the left panel of Fig. 1. Without loss of generality, we fix $\mathbf{p}$ on the z axes, $\mathbf{k}$ on $x-z$ plane. The angle between $\mathbf{p}$ and $\mathbf{k}$ is θ and the angle between $\mathbf{a}$ and $\mathbf{p}-\mathbf{k}$ plane is ϕ.

Considering the ultra relativistic case, a boost along the $(\hat{e}_\mathbf{k}+\hat{e}_\mathbf{p})/2$ direction with $\beta=\cos\frac{\theta}{2}$ transforms $\mathbf{k}$ and $\mathbf{p}$ antiparallel. In the boosted frame, apply Eq. (20) and Eq. (21),

$$\begin{aligned} k'^B_0 &\approx \frac{u(1+C)}{1+u}p^B_0 \\ p'^B_0 &\approx \frac{1+u^2C}{1+u}p^B_0 \\ \cos\theta^B_{k'} &\approx \frac{1-C}{1+C} \\ \cos\theta^B_{p'} &\approx \frac{1-u^2C}{1+u^2C}, \end{aligned} \tag{22}$$

where the superscript B denotes the boosted frame and $p^B_0=\sin\frac{\theta}{2}p_0$.

Apply the reverse lorentz boost, one gets

$$\mathbf{p}'_\pm \approx \frac{(1+u^2C)p_0}{1+u} \begin{pmatrix} \sin\theta\sin^2\frac{\theta^B_{p'}}{2}\pm\sin\frac{\theta}{2}\cos\phi\sin\theta^B_{p'} \\ \mp\sin\frac{\theta}{2}\sin\theta^B_{p'}\sin\phi \\ 1-2\sin^2\frac{\theta^B_{p'}}{2}\sin^2\frac{\theta}{2}\pm\cos\phi\cos\frac{\theta}{2}\sin\theta^B_{p'} \end{pmatrix} \tag{23}$$

and

$$\mathbf{k}'_\pm \approx \frac{u(1+C)p_0}{1+u} \begin{pmatrix} \sin\theta\sin^2\frac{\theta^B_{k'}}{2}\mp\sin\frac{\theta}{2}\cos\phi\sin\theta^B_{k'} \\ \pm\sin\frac{\theta}{2}\sin\theta^B_{k'}\sin\phi \\ 1-2\sin^2\frac{\theta^B_{k'}}{2}\sin^2\frac{\theta}{2}\mp\cos\phi\cos\frac{\theta}{2}\sin\theta^B_{k'} \end{pmatrix} \tag{24}$$

where the $\pm$ subscripts are the sign ρ and the chances for both signs are 1/2 for the differential probability is a even function of ρ.

Since NBW is the symmetric process of NCS, deduction for the involved laser photon number in a single NBW in UILs is very similar, which gives

$$n^{CCF}_{NBW} \approx \frac{1}{\delta(1-\delta)\chi'}a_0^3 \tag{25}$$

when $a_0 \gg 1$, where the parameter δ and χ' are given in the paper.

Equations for single NBW is also similar:

$$\begin{cases} nk+k'' = p''+p''' \\ \delta = \dfrac{kp''}{kk'} \\ n = \dfrac{1}{\delta(1-\delta)\chi'}a_0^3 \\ \tau/a_0 = 0 \end{cases}. \tag{26}$$

Solve these equations in the frame shown in the right panel of Fig. 1, one obtains

$$\mathbf{p}''_\pm \approx \frac{(k''_0+nk_0)}{2}(1+(2\delta-1)\zeta) \begin{pmatrix} \sin\theta\sin^2\frac{\theta^B_{p''}}{2}\mp\sin\frac{\theta}{2}\cos\phi\sin\theta^B_{p''} \\ \pm\sin\frac{\theta}{2}\sin\theta^B_{p''}\sin\phi \\ 1-2\sin^2\frac{\theta^B_{p''}}{2}\sin^2\frac{\theta}{2}\mp\cos\frac{\theta}{2}\cos\phi\sin\theta^B_{p''} \end{pmatrix}, \tag{27}$$

and

$$\mathbf{p}'''_\pm \approx \frac{(k''_0+nk_0)}{2}(1-(2\delta-1)\zeta) \begin{pmatrix} \sin\theta\sin^2\frac{\theta^B_{p'''}}{2}\pm\sin\frac{\theta}{2}\cos\phi\sin\theta^B_{p'''} \\ \mp\sin\frac{\theta}{2}\sin\theta^B_{p'''}\sin\phi \\ 1-2\sin^2\frac{\theta}{2}\sin^2\frac{\theta^B_{p'''}}{2}\pm\cos\phi\cos\frac{\theta}{2}\sin\theta^B_{p'''} \end{pmatrix}, \tag{28}$$

where

$$\begin{aligned} \cos\theta^B_{p''} &= \frac{\zeta+(2\delta-1)}{1+(2\delta-1)\zeta} \\ \cos\theta^B_{p'''} &= \frac{\zeta-(2\delta-1)}{1-(2\delta-1)\zeta}, \end{aligned}$$

and

$$\zeta = \frac{k''_0-nk_0}{k''_0+nk_0}. \tag{29}$$

Again, $\pm$ subscripts are the sign of ρ' and both signs take half of the chance.

[1] V. I. Ritus, J. Sov. Laser Res. **6**, 497 (1985).
[2] C. Harvey, T. Heinzl and A. Ilderton, Phys. Rev. A, **79**, 063407 (2009).
[3] V. B. Berestetskii, E. M. Lifshitz, L. P. Pitaevskii, *Quantum Electrodynamics* 2nd Edition, (1999) Elsevier (Singapore).
[4] D. Seipt, T. Heinzl, M. Marklund, and S. S. Bulanov, Phys. Rev. Lett. **118**, 154803 (2017).
